# Thermal Effects of Dental Filling in Teeth

**Published:** 1865-08

**Authors:** H. A. Smith


### THERMAL EFFECTS OF METAL FILLING IN TEETH

BY H. A. SMITH, D. D. S.

Read before the American Dental Convention, at White Sulphur Springs, Aug. 1, 1865.

The introduction of metallic fillings into cavities of decay in teeth is frequently followed by more or less pain and discomfort to the patient, and when certain conditions are present seriously endangers the vitality of the tooth. This is due mainly to the high conducting power of the metals used for this operation; though in this respect they differ widely.

It is my purpose in this paper to give the results of some experiments with the metals and amalgams usually employed in this operation, with a view to ascertain their thermal effects upon sensitive dentine. Also to examine the relative value of the various substances used as non-conductors to prevent the ill effects referred to.

In this discussion it will be observed that I have adopted the dynamical, or as it is frequently called the *mechanical theory* of heat, or that the essence of heat is *motion*.

*Temperature* exercises a powerful influence over the animal functions, stimulating them into activity or depressing them as it is increased or diminished. The extremes of heat or cold, that man and the higher animals can subject themselves to, is truly wonderful. Experiments have shown that eggs can be boiled by the heat of an oven, in which the living bodies of men are uninjured. When heat is communicated gradually to the body, or an organ of the body, its temperature is scarcely increased. The blood of the inhabitant of the frigid zone does not differ sensibly in temperature from that of one living in a hot climate. If the temperature of the air surrounding the body is increased in a gradual manner, it serves only to quicken the vital activity. The heat instead of being used to increase the temperature of the body, is consumed in the work of preparing the perspiration, and driving it to the surfaces. All the animal functions are quickened. The heat being consumed in this work, there is no sensible increase in the blood temperature.

Now, when a tooth is filled with a metal, and hot substances are brought in contact with the surface of the plug, the sensation felt depends in degree upon the slowness or rapidity with which the heat is transmitted through the metal. If it is conveyed to the pulp in a gradual manner, the functions of that organ are simply increased. The heat is consumed in work, and no inconvenience is felt.

If the tooth is in a normally healthy condition, any of the metals used may be safely introduced. If, however, a sensitive condition of the dentine exists, a non-conducting material is resorted to, which is placed between the metal and sensitive dentine. This low-conducting body does not prevent the passage of the heat through it. It only accumulates it upon its surface, and the heat is transmitted so slowly, that it can be used up in the work of quickening the vital energy of the pulp. In a similar manner we are effected if we take hold alternately with the hand, articles of iron, wood and marble, subject to the same source of heat. All seem to us of a different temperature, when in fact they are precisely the same. If the articles touched with the hand are warmer than the body, the iron will feel warmer than the marble, the marble warmer than the wood. The heat is communicated to the body by the poorer conductor in so gradual a manner that an increase of temperature is hardly felt.

The philosophy of employing non-conducting substances for temporary fillings will now be readily understood. Or if a metal is used, the importance of choosing one which has a low conducting power, with a high capacity for heat.

The following table shows the relative conductive powers of the several metals used for fillings :

| NAME. | CONDUCTIVITY. |
|---|---|
| Silver | 100 |
| Gold | 60 |
| Fusible Metal | 54 |
| Amalgam | 49 |
| Tin | 17 |
| Lead | 10 |

These numbers show how widely these metals differ in conductibility.

Taking the amount of heat given out by a pound of water in cooling one degree of temperature, as unity, the following numbers it has been found express the specific heat of the metals enumerated:

| | |
|---|---|
| Water | 1,0000 |
| Silver | 0,0570 |
| Gold | 0,0330 |
| Fusible Metal | 0,0420 |
| Amalgam | 0,0338 |
| Tin | 0,0562 |
| Lead | 0,0308 |
| Iron | 0,1138 |
| Bismuth | 0,0308 |

Lead is frequently spoken of as a much more desirable filling for a sensitive cavity than tin. If their capacity for heat was the same, the difference in their conductibility would be greatly in favor of lead. But when by reference to the table, we find that the specific heat of lead is little more than one-half that of the tin, it is very doubtful if the lead should be given any preference over the tin in this particular. Tin has a high capacity for heat with low conductivity. Gold on the contrary has a high conducting power with a low specific heat, and it is for this reason that tin has so decided a preference over gold, as a filling considered in its thermal effects upon the tooth.

To better illustrate the relation of specific heat to the conducting power of a metal, let us suppose that we have filled the same cavity in a tooth alternately with iron and bismuth, and apply the same amount of heat to each of them. The high conductivity of iron compared with bismuth, would indicate that the sensation of heat would be soonest felt through the iron. Iron 12 bismuth 2. But such would not be the case. The bismuth would first communicate the heat to the nerves of sensation. This apparent paridoxical result is explained by the great difference in the capacity for heat of the two metals. Iron 0,1138, bismuth 0,0308. The iron requires three times the quantity of heat to raise it to the same degree of temperature that the bismuth does. The iron, though much the best conductor, yet owing to the difference in the number of its atoms, or the great amount of interior work performed, does not quickly increase in temperature. On the other hand the bismuth at once uses a large share of the heat applied, to raise its temperature, and thus we find the result as stated.

Ordinarily if the dentine is in a normal condition, and any considerable thickness of dentine is left in the bottom of the cavity no inconvenience is felt if a metal filling is introduced. The dentine is a much poorer conductor than the metals used, and the heat conducted down through the filling is in a gradual manner conveyed to the pulp. In those cases where a sensitive condition of the dentine exists, or only a very thin lamina of bone covers the pulp, it is often practicable to use some non-conducting substance to place between the filling and the bottom of the cavity.

In organic substances the transmissive power for heat is exceedingly low, and we naturally look to these for material to be used for this purpose.

The following table gives the results of some experiments to ascertain the relative conducting powers of some of the organic substances used for non-conductors.

The standard or unity is applied to the substance found to conduct heat most readily of any experimented with, viz: *oiled silk.*

| | |
|---|---|
| Oiled Silk - - - - - | 1,00 |
| Asbestos - - - - - | ,52 |
| Paper (Bristol Board) - - - | ,43 |
| Wood (length of fiber) - - - | ,38 |
| Do. (across do. ) - - - | ,36 |
| Quill - - - - - - - | ,32 |
| Spunk - - - - - - - | ,25 |
| Bark of Wood (across fiber) - - | ,23 |
| Do. do. (length do. ) - - | ,25 |

In comparison with these dentine has a conductivity of ,40.

The mechanical state of bodies influences greatly their heat transmissive powers. The soft light substances mentioned in the table as asbestos and spunk, may, when subject to the pressure required for a good filling show very much higher conductivity. The motion of heat is greatly retarded through a fibrous substance by the air filling the spaces. The tubular structure of dentine doubtless does much to prevent the passage of heat through it. Experiments show wood and dentine to have about the same conductivity, wood 38, dentine 40. These experiments were made of course upon dentine deprived of vitality. The comparative conductivity of living and dead dentine can only be conjectured. Though it is probable that it will conduct as well with the tubules filled with fluids, as when these are dried up, as they would be in the dead tissue, and the tubules filled with air.

Having ascertained the conductive power of dentine, the thickness of bone covering the pulp would apparently indicate whether or not a non-conductor should be placed under the filling. If the layer of bone was deprived of its vitality, or the tooth an inorganic substance, none would be needed. But in practice we find such is not the fact.

Accepting the *hypothesis* that the dentinal tubules are filled with *nerve fibrils* it is not necessary that the heat should be communicated directly to the pulp, before a sensation of pain is felt. The impression is made directly on the sensory nerves in the dentine, and by them conveyed to the sensorium through the pulp. Covering the dentine with a non-conducting substance prevents the heat passing more rapidly than can be used in the work of quickening the vital action of the tooth.

The practice of only protecting the layer of bone covering the pulp it will be seen, is not sufficient. Dentine conducts heat in all directions nearly alike, and sensations sufficient to disturb the pulp, may be conveyed from a point remote from it in a cavity.

The practice of leaving a portion of decayed bone in the bottom of the cavity and filling over it, has been strongly advocated by some members of the profession. Without reference to the pathological results of the practice, let us look at it in its thermal effects.

The fact that in many cases, no pain was felt in the tooth until the cavity had been excavated and filled with a metal indicates that the carious bone and the foreign matter usually found in cavities of decay are poor conductors of heat. This foreign matter is of animal or vegetable origin. We have seen how slowly vegetable substances conduct heat in the example of *wood*, and animal matter, as the muscle of flesh has a much lower conducting power. A cavity in which such accumulations were found would not ordinarily be effected by a change of temperature, unless the pulp was actually exposed. It is only when this *debra* has been removed that the tooth is sensitive to change of temperature.

The chemical nature of the carious bone remaining in the cavity would modify the facility with which heat is conducted through it. If it is of the kind denominated the soft variety of decay, the remaining portion is nearly all of the organic or animal constituents of the dentine, it is natural to suppose that the conductive power would be less than if the inorganic only remained. It has been ascertained that rock chrystal compared with the muscle of flesh, conducts in the ratio of 100 to 1. All are familiar with the fact that the incrustation upon kettles in which hard water is boiled seriously prevents the passage of the heat to the water. This deposit in many cases is carbonate of lime, one of the constituents of tooth bone. If in the solid form, as in this incrustation, it is so low a conductor, in the divided condition, in which we find the salts of lime in this variety of decay, they would certainly have a much lower conductivity.

If this be true, then, leaving a portion of carious bone, whether of the hard or soft variety of decay, would materially intercept the passage of heat to the sensitive parts beneath, and for this purpose the practice would be a good one.

I have in this paper used the term *conduction* as applied to the manner in which heat is conveyed through the dentine to the pulp, and have regarded the pulp as the medium directly through which the sensation is felt. The question suggests is this strictly true. If the dentinal tubes contain nerve fibrils, this cannot be the case, for when the heat has been conducted through the metal, the instant the motion is imparted to the nerve fibrils in the dentine, the impression is made directly upon them, and thus conveyed through the pulp to the brain.

If no nerve filiments are to be found in the dentine, and the tubules are filled with *liquor sanguines*, the sensation could more properly said to be *conducted* to the pulp. Heat expands all bodies. This implies a motion of the particles of the body expanding, reasoning thus—*the essence of heat is motion*, and this motion is propagated through the dentine, and imparted to the pulp, thence the impression is carried to the seat of sensation. In the one instance the nerves of sensation are in contact with the hot body the plug, and the sensation is at once conveyed through them to the pulp. In the other the impression is not made directly upon the nerve, but is rather *conducted* through the dentine to the pulp and thence to the sensorium. Thus the motion of heat is the cause of any impression that may be induced by a change of temperature.

It will be observed that no reference has been made to the effects of cold when brought in contact with a metal filling.

The effects of intense heat and intense cold are the same. A hot iron will not more quickly blister the skin than frozen mercury. Heat and cold are merely relative. Cold is the absence of heat. When the metal plug is chilled, we simply have the motion reversed. The cold is not properly conducted to the tooth, but the heat is conducted from the hot body—the tooth—to the cold metal, and this continues till they are both of the same temperature.

